# A United States Fair Lending Perspective on Machine Learning

**DOI:** 10.3389/frai.2021.695301

**Published:** 2021-06-07

**Authors:** Patrick Hall, Benjamin Cox, Steven Dickerson, Arjun Ravi Kannan, Raghu Kulkarni, Nicholas Schmidt

**Affiliations:** ^1^ The George Washington University, Washington, DC, United States; ^2^ BNH.ai, Washington, DC, United States; ^3^ H2O.ai, Mountain View, CA, United States; ^4^ Discover Financial Services, Riverwoods, IL, United States; ^5^ BLDS, LLC, Philadelphia, PA, United States; ^6^ Solas.ai, Philadelphia, PA, United States

**Keywords:** credit underwriting, fairness, interpretability, XAI (explainable artificial intelligence), deep learning—artificial neural network (DL-ANN), evolutionary learning, Shapley values, machine learning

## Abstract

The use of machine learning (ML) has become more widespread in many areas of consumer financial services, including credit underwriting and pricing of loans. ML’s ability to automatically learn nonlinearities and interactions in training data is perceived to facilitate faster and more accurate credit decisions, and ML is now a viable challenger to traditional credit modeling methodologies. In this mini review, we further the discussion of ML in consumer finance by proposing uniform definitions of key ML and legal concepts related to discrimination and interpretability. We use the United States legal and regulatory environment as a foundation to add critical context to the broader discussion of relevant, substantial, and novel ML methodologies in credit underwriting, and we review numerous strategies to mitigate the many potential adverse implications of ML in consumer finance.

## Introduction

Within the financial services industry, lenders’ use of machine learning (ML) to measure and identify risk in the provision of credit can benefit both financial institutions (FIs) and the consumers and businesses that obtain credit from lenders. FIs generally have strong guardrails in place for model development, validation, and audit that, if appropriately designed, can help minimize the inherent risks associated with ML technologies (such as discrimination, privacy, security, and other risks). A robust regulatory regime already mandates transparency, nondiscrimination, and stability for predictive models.^1^ Because this governance process is generally extensively prescribed, changing it to adopt new technology can be slow and arduous. In this mini review, we first establish uniform definitions of key ML concepts so that market participants, regulators, policymakers, and other stakeholders can communicate effectively when moving toward the adoption of ML. To provide a realistic portrayal of the additional governance required when deploying ML, this mini review then describes current best practices for mitigating ML-related harms with controls aligned to the current United States legal and regulatory environment.

## Definitions

Establishing a common language is essential for stakeholders to discuss issues productively. Since ML is an evolving field of computational science, new vocabulary arises in the public dialogue including terms and phrases that may, or may not, be relevant to the practice of ML in lending. To further adoption discussions, this section provides specific, uniform definitions for key concepts.

### Discrimination: Protected Classes and Legal Standards

Since the 1960s, United States laws have prohibited illegal discrimination and have evolved over time to establish a strong framework for safeguarding the rights of certain groups of consumers that have been historically disadvantaged and thus deemed “protected classes.”^2^ For example, the Equal Credit Opportunity Act (ECOA), enacted in 1974, prohibits illegal discrimination in any aspect of a credit transaction based on an applicant’s race, color, religion, national origin, sex, marital status, or age, as well as other “prohibited bases.” Similarly, the Fair Housing Act (FHA) prohibits illegal discrimination in the mortgage lending or housing context.^3^

Discrimination perpetuated by shoddy ML models is often encoded in data long before algorithms are trained (Hassani, 2020), but biases can also arise from poor experimental design and other phenomenon not directly associated with ML model mechanisms. However, compliance efforts relating to mitigation of any illegal discrimination in lending tend to focus on modeling outcomes. Two theories of liability under ECOA and FHA for discrimination against members of protected classes are “disparate treatment” and “disparate impact.”^4^ Below we outline commonly accepted definitions of these terms and their relationship to ML.

**Disparate Treatment:** Disparate treatment occurs when a lender treats an applicant differently based on one of the prohibited bases (e.g., race or sex) in any aspect of a credit transaction, including the provision of credit and setting of credit terms (e.g., pricing). Disparate treatment discrimination is always illegal in lending in the United States and does not require any showing that the treatment was motivated by prejudice or a conscious intent to discriminate.

**Disparate Impact:** Disparate impact occurs when a lender employs a neutral policy or practice equally to all credit applicants but the policy or practice disproportionately excludes or burdens certain persons on a prohibited basis. Disparate impact is not necessarily a violation of law and may be justified by a business necessity, such as cost or profitability, and by establishing there is no less discriminatory alternative to the policy, practice, or model (See text footnotes^5^).

### Explanation, Interpretable Models, and Scope Definitions

Transparency into the intricacies of ML systems is achieved today by two primary technical mechanisms: directly interpretable ML model architectures and the post hoc explanation of ML model decisions. These mechanisms are particularly important in lending, because under ECOA’s implementing regulation, Regulation B, and the Fair Credit Reporting Act (FCRA), the principal reasons for many credit decisions that are adverse to the applicant must be summarized to consumers through a set of short written explanations known as “adverse action notices.”

**Adverse Action Notices:** Under Regulation B, lenders must notify an applicant in writing of the principal reasons for taking an adverse action on a loan application within a specific time period.^6^ When using ML systems to make credit decisions, the principal reasons included on adverse action notices are explanations based on ML system input features that negatively affected the applicant’s score or assessment.

Regulation B provides standards for the factors lenders may use and how lenders must inform applicants of credit decisions based on credit scoring models.^7^ For a rejected application, lenders must indicate the principal reasons for the adverse action and accurately describe the features actually considered. The notice must include a specific considered input feature but is not required to state how or why this feature contributed to an adverse outcome. Crucially, adverse action notices are also part of a broader framework that enables actionable recourse for consumer decisions based on inaccurate data.

**Interpretability and Explainability:** Finale Doshi-Velez and Been Kim define interpretability as “the ability to explain or to present in understandable terms to a human” (Doshi-Velez and Kim, 2017). Professor Sameer Singh of the University of California at Irvine defines an explanation in the context of an ML system as a “collection of visual and/or interactive artifacts that provide a user with sufficient description of a system’s behavior to accurately perform tasks like evaluation, trusting, predicting, or improving a system” (Hall et al., 2019). “Interpretable” usually describes directly transparent or constrained ML model architectures, and “explanation” is often applied to a post hoc process that occurs after model training to summarize main drivers of model decisions^8^. Both concepts are important for adverse action notice reporting, because the more interpretable and explainable an ML system, the more accurate and consistent the associated adverse action notices.

**Global and Local Scope:** A closely related concept to explanation is “scope.” ML systems can be summarized “globally” (across an entire dataset or portfolio of customers) and “locally” (for only a subset of a dataset or a smaller group of customers, including a single customer). Both global and local explanations are important to FIs when deploying ML. Global explanation results are often documented as part of an FI’s model governance processes to meet regulatory standards on model risk management,^9^ while local customer-specific explanations are likely to be a primary technical process behind adverse action notice generation for FCRA and ECOA compliance.

## Considerations Around Discrimination

There are many ways that analysts and data scientists can define and mitigate discrimination in ML (Barocas et al., 2018).^10^ However, only a subset of the discrimination measurement and mitigation techniques available today are likely to be appropriate for fair lending purposes. This subsection describes a few established discrimination measurements, discusses some newer measures and mitigation techniques, and explains why, if some newer approaches are used, fair lending regulations must be carefully considered in order to properly mitigate noncompliance risk.

### Traditional Methods for Identifying Discrimination

Given the recent interest in fairness and ethics in ML, it may appear that algorithmic discrimination is a new issue. On the contrary, testing outcomes in education, lending, and employment for discrimination is a decades-old discipline (Hutchinson and Mitchell, 2019). For example, marginal effect (ME)^11^ provides one way to measure disparate impact in ML lending models. Other techniques, such as the adverse impact ratio (AIR)^12^ and the standardized mean difference (SMD, which is also known as “Cohen’s d”) (Cohen, 1988), which have a long history of use in employment discrimination analyses, can also be used for measuring disparate impact in lending.

### Recently Proposed Discrimination Definitions and Discrimination Mitigation Techniques

Over recent years, ML and fair lending experts have explored ways to measure and mitigate discrimination in ML. These discrimination mitigation techniques come in three forms: pre-processing, in-processing, and post-processing. Pre-processing techniques [e.g., reweighing (Kamiran and Calders, 2012)] diminish disparities in the data used to train ML models. In-processing methods [e.g., adversarial de-biasing (Zhang et al., 2018)] are ML algorithms that themselves remove disparities from their predictions as the models learn. Post-processing techniques [e.g., reject option classification (Kamiran et al., 2012)] change the predictions of an ML model in order to minimize discrimination.^13^

### Regulatory Compliance

It is imperative that FIs employ the appropriate use of discrimination testing and mitigation methods for regulated applications in fair lending because some methods may lead to counterproductive results or even to noncompliance with anti-discrimination statutes.

It is difficult to optimize on multiple metrics of fairness at one time—there is necessarily a trade-off where making a model fairer by one measure often makes it appear less fair by another (See text footnote 10). While academic literature on ML fairness has focused on balancing error rates, regulators and courts have generally focused on minimizing differences in favorable outcomes between groups, regardless of the underlying distribution of true outcomes within each group. Certain open source and commercially available software have followed the academic practice and focused on measures of relative error rates.^14^ While these are important and often useful measures of fairness, if a lender were to choose among models based on error rates alone, then they may cause traditional measures of disparate impact, such as ME (discussed above), to become worse. Therefore, focusing on more traditional discrimination measures may be the safer route for practitioners in fair lending.

Similar scenarios can also arise for other potential discrimination mitigation techniques. Since ECOA generally prohibits the use of protected class status when making a lending decision—arguably even if the lender intends to use it to make its lending decisions fairer—discrimination mitigation methodologies that require explicit consideration of protected class status in training or inference are unlikely to be considered acceptable. In fact, because FIs are explicitly prohibited from collecting information such as race and ethnicity (apart from mortgage lending), these techniques may also simply be infeasible.

**FIGURE 1:**
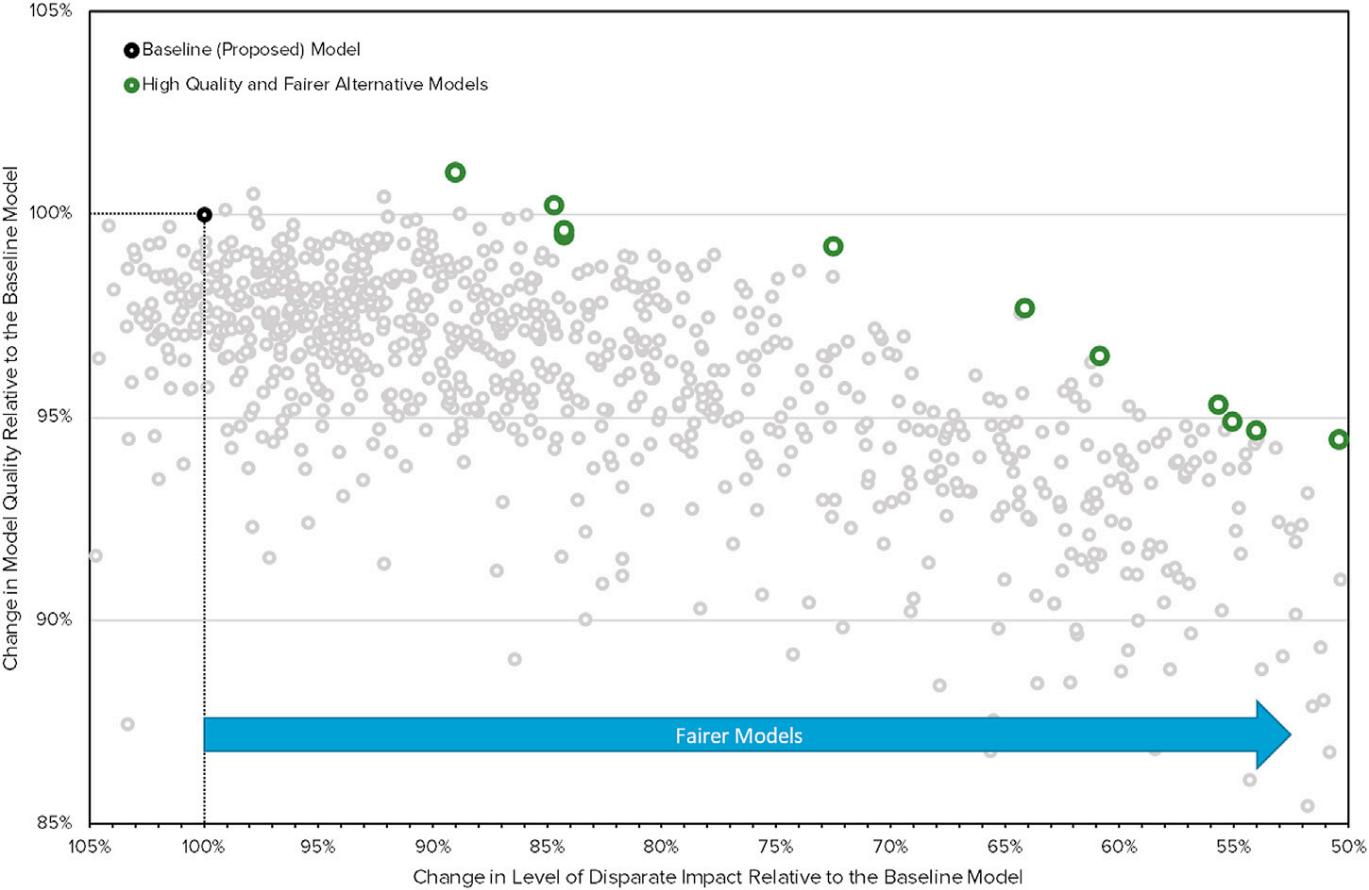
Results from a multi-objective evolutionary search procedure in which several lending models are found that retain the performance quality, but decrease disparate impact, in a realistic lending model.

Given such restrictions, mitigation approaches that perform feature selection and hyperparameter searches may be considered natural extensions of traditional approaches and are likely to be subject to less concern. Figure 1 presents the results of a multi-objective evolutionary search procedure in which several lending models are found that retain the performance quality and decrease disparate impact of an example lending model, without the inclusion of protected class information in any considered models. Using results like those in Figure 1, FIs can select the most accurate (highest on *y*-axis) and least discriminatory (rightmost on *x*-axis) model that meets business and legal requirements. Other methods that do not rebalance data or decisions and that do not explicitly consider protected class status may gain wider acceptance as they are used more frequently and are shown to be effective ways to decrease discrimination (Miroshnikov et al., 2020).

## Considerations Around Transparency

Like discrimination testing and mitigation approaches, many new techniques for understanding ML models have been introduced in recent years, which can create both transparent models and summaries of model decisions. They are already being used in the financial services industry today^15^ and are likely to be deployed for lending purposes. This subsection introduces some of these techniques and important considerations for their use in lending.

### Examples of Interpretable Machine Learning Models

In the past, ML researchers and practitioners operated under what appeared to be a natural trade-off: the more accurate a model, the more complex and harder to understand and explain. Today, the landscape has changed for predictive modelers in credit lending with the advent of highly accurate and highly interpretable model architectures that appear to break the so-called “accuracy-interpretability trade-off.” In fact, some leading scholars have posited that for structured tabular data used most commonly in lending models, black boxes are likely not more accurate than interpretable ML models (Rudin, 2019).^16^ Interpretable ML models include: variations of linear models [e.g., explainable boosting machines (EBMs, also known as GA2Ms) (Lou et al., 2013)]; constrained tree-based models [e.g., optimal sparse decision trees (OSDTs) (Hu et al., 2019), monotonic gradient boosting machines (MGBMs)^17^]; constrained neural networks [e.g., Explainable Neural Networks (XNNs) (Vaughan et al., 2018)]; novel or constrained rule-based models [e.g., scalable Bayesian rule lists (SBRLs) (Yang et al., 2017) and CORELS (Angelino et al., 2018)]; and several others. Levels of interpretability vary from results understood only by advanced technical practitioners (e.g., MGBMs or XNNs), to results that business and legal partners could likely consume directly (e.g., OSDTs or SBRLs), to something in-between (e.g., EBMs). Beyond their obvious advantages for adverse action notice requirements, interpretable ML models may also assist practitioners in model governance and documentation tasks, such as understanding which input features drive model predictions, how they do so, and which feature behavior under the model aligns with human domain knowledge. Moreover, interpretable models may help in discrimination testing and remediation by transparent weighting and treatment of input features.

### Examples of Post Hoc Explanations

Post hoc explanation techniques create summaries of varying types and accuracy about ML model behavior or predictions. These summaries can provide an additional, customizable layer of explanation for interpretable ML models, or they can be used to gain some amount of insight regarding the inner workings of black-box ML models. Summary explanations can have global or local scopes, both of which are useful for adverse action notice generation, model documentation, and discrimination testing. Post hoc explanations can be generated through numerous approaches, including direct measures of feature importance [e.g., gradient-based feature attribution (Ancona et al., 2018), Shapley values (Roth, 1988; Lundberg and Lee, 2017)], surrogate models [e.g., decision trees (Craven and Shavlik, 1996; Bastani et al., 2019), anchors (Ribeiro et al., 2018), local interpretable model-agnostic explanations (LIME) (Ribeiro et al., 2016)], and plots of trained model predictions [e.g., accumulated local effects (ALE) (Apley and Zhu, 2019), partial dependence (Friedman et al., 2001), and individual conditional expectation (ICE) (Goldstein et al., 2015)].

### The Importance of Dual Scope Explanations

An important and often discussed aspect of ML interpretability is the scope of an explanation—whether an explanation is local or global. Many new research papers focus on local explanations for evaluating the impact of a feature at the individual customer level. However, seeing both a global and local view presents a more holistic picture of model outcomes. For example, it is important for actionability that a customer understand which global factors resulted in an adverse action on their credit decision (e.g., length of credit history), while also understanding the local factors that are within their control to achieve a favorable outcome in the near future (e.g., lower utilization of credit limit).

### Grouping Correlated Features for Explanation

Many explanatory techniques are less accurate in the presence of correlated input features (Altmann et al., 2019; Kumar et al., 2020) as post hoc explanation methods do not often account for dependencies between features. Grouping involves treating a group of correlated features (with strong correlations between features in the group and weak correlations with features outside of the group) as one from an explanation standpoint. Grouping can help produce consistent explanations by unifying conditional and marginal explanations (Miroshnikov et al., 2021) potentially addressing a point of contention in the recent literature (Frye et al., 2020; Janzing et al., 2020). By increasing the fidelity of post hoc explanations and summarizing larger numbers of input features, grouping may also help provide more meaningful adverse action notices to customers.

### Additional Concerns for Post Hoc Explanations

Like all other ML techniques post hoc explanation approaches have pros and cons, and they should never be regarded as a perfect view into complex model behaviors. Well-known pitfalls include partial dependence failing in the presence of correlated or interacting input features, inaccurate surrogate models that do not truly represent the underlying complex model they seek to summarize, and inconsistencies in feature importance values (Altmann et al., 2019; Hall, 2020; Kumar et al., 2020). This subsection will briefly outline some of the most fundamental issues for post hoc explanations: inconsistency and problems with human comprehension of explanations.

#### Inconsistent Explanations

**FIGURE 2:**
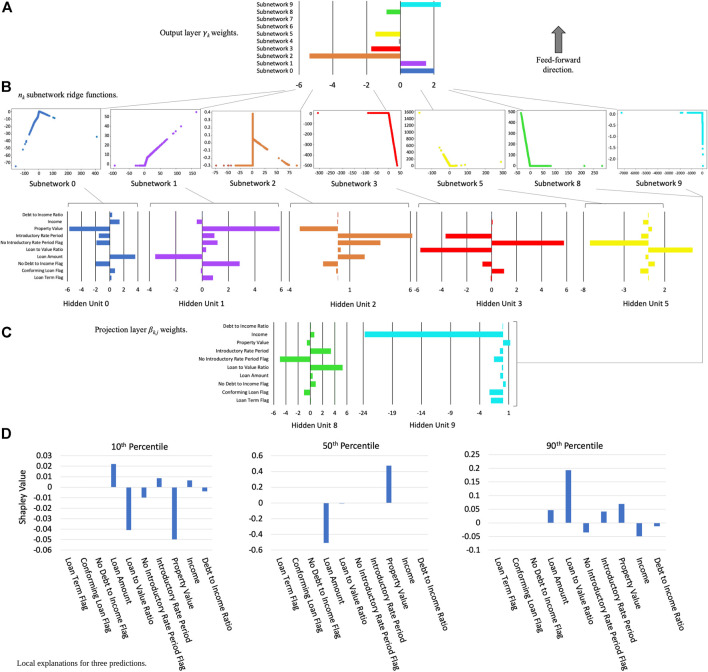
An XNN trained to predict high-priced loans from publicly available mortgage data and local Shapley feature contributions for three specific customer predictions (Vaughan et al., 2018): **(A)**. XNN output layer—global weighting of *n*_*k*_ subnetworks for XNN output; **(B)**. Internal subnetworks—*n*_*k*_ subnetworks associated with a specific weighting in projection layer *β*_*k,j*_ weights; **(C)**. Projection layer—input layer weights that project data into the next layer of the XNN; and **(D)**. Shapley value local feature contributions for customers at the 10th, 50th, and 90th percentiles of model output, calculated with the DeepSHAP method (Lundberg and Lee, 2017; Gill et al., 2020).

Since many ML explanation techniques are inconsistent, different ML models, different configurations of the same ML model, or refreshing the same ML model with new data can result in different explanations for the same consumer if not controlled. Inconsistency bears special consideration in financial services, especially for the generation of adverse action notices for credit lending decisions, where two similar models giving different explanations to the same applicant may raise questions, if not lead to regulatory noncompliance or reputational harm for the FI. To mitigate risks associated with inconsistent explanations, FIs may consider pairing post hoc explanations with constrained and stable ML models, explicitly test for explanation stability, group correlated features where appropriate for explanation, and/or consider using explanation techniques with consistency guarantees (Miroshnikov et al., 2021). As an example of pairing constrained models with consistent explanations, Figure 2 displays the global architecture of an XNN model and associated Shapley value contributions for several customer predictions on publicly available mortgage data. (Figure 2 also provides an example of how so-called black-box models can be re-architected and constrained to create transparent models that are more amenable to debugging by technical practitioners).

#### Human Comprehension of Explanations

Concerns have been raised that nontechnical audiences (e.g., most credit applicants) cannot easily understand ML models and explanations (Tomsett et al., 2018; Kumar et al., 2020; Poursabzi-Sangdeh et al., 2021). In financial services, there are several less technical audiences to consider, including validation and audit personnel, business partners, legal and compliance professionals, and consumers. The success of an explainable ML project often hinges on the comprehension of model behavior by less technical audiences, and specific user-interaction modalities must be considered during system design, implementation, and deployment.

### Explanations for Discrimination Testing

Recent work has shown that explanation techniques can be used to guide an understanding of both the discriminatory and predictive impacts of each feature (Miroshnikov et al., 2020).^18^ With this information, a model builder can structure a search for alternative models more efficiently *by removing* features with low importance and large contributions to disparate impact, and *by including* important features that contribute less toward disparate impact. In combination with ever-increasing computing resources, the ability to apply explanation techniques to understand specific drivers of disparate impact makes testing a large number of possible alternative models feasible, enabling model builders and compliance professionals to perform a more robust search for less discriminatory models that maintain their predictive ability (Schmidt and Stephens, 2019; Schmidt et al., 2021).^19^

### Regulatory Compliance

ECOA and Regulation B do not prescribe a specific number of adverse action notices to share with consumers, nor do they prescribe specific mathematical techniques.^20^ However, regulatory commentary indicates that more than four reasons may not be meaningful to a consumer. FIs also have flexibility in selecting a method to identify principal reasons. Regulation B provides two example methods for selecting principal reasons from a credit scoring system but allows creditors the flexibility to use any method that produces substantially similar results. One method is to identify the features for which the applicant’s score fell furthest below the average score for each of those features achieved by applicants whose total score was at or slightly above the minimum passing score. Another method is to identify the features for which the applicant’s score fell furthest below the average score for each of those features achieved by all applicants. Both examples appear to be generally aligned with high quality Shapley value, gradient-based, or counterfactual explanations (Wachter et al., 2017), and interpretable ML models. Such technologies can also assist in compliance with model documentation requirements.

## Conclusion

This mini review provides a simplified, yet substantive, discussion of key definitions and considerations for using ML within the United States lending context. While questions remain as to which methods will be most useful for ensuring compliance with regulatory requirements, variants of constrained models, Shapley values, and counterfactual explanations appear to be gaining some momentum in the broader lending community (Bracke et al., 2019; Bussman et al., 2019). From the fair lending perspective, there are well-established discrimination testing and mitigation methodologies that have been used for decades. Fair lending practitioners must now work with legal and compliance colleagues to leverage recent ML advances without unintentionally violating existing regulatory and legal standards. Of course, discrimination and interpretability are only two of many concerns about ML for first-, second-, and third-line personnel at United States FIs. As models become more sophisticated and FIs become more dependent upon them, and as data privacy and artificial intelligence (AI) regulations grow in number and complexity—as exemplified by the recent E. U. proposal for AI regulation and increased saber-rattling by the United States Federal Trade Commission (FTC), proper model governance, and human review, and closer collaboration between legal, compliance, audit, risk, and data science functions will likely only increase in importance.

## Footnotes

1 E.g., The Equal Credit Opportunity Act (ECOA), The Fair Credit Reporting Act (FCRA), The Fair Housing Act (FHA), and regulatory guidance, such as the Interagency Guidance on Model Risk Management (Federal Reserve Board, SR Letter 11–7). The E. U. Consumer Credit Directive, Guidance on Annual Percentage Rates (APR), and General Data Protection Regulation (GDPR) serve to provide similar protections for European consumers.

2 Civil rights legislation has a much longer history in the United States, beginning with the 1866 Civil Rights Act (ratified in 1870), passed in the wake of the United States Civil War. Modern civil rights legislation, beginning in earnest in the 1960s, initially focused on employment but has been extended to provide broader protections, including in areas such as credit and housing. Non-discrimination in the E. U. is enshrined in Article 21 of the Charter of Fundamental Rights, Article 14 of the European Convention on Human Rights, and in Articles 18–25 of the Treaty on the Functioning of the E. U.

3 Prohibited bases under FHA include race, color, religion, national origin, sex, familial status, and disability. The GDPR, as an example of non-United States regulation, prohibits use of personal data revealing racial or ethnic origin, political opinions, and other categories somewhat analogous to protected bases.

4 See CFPB Supervision and Examination Manual, Pt. II, §C, *Equal Credit Opportunity Act* (Oct. 2015).

5 The United States Supreme Court established the disparate impact theory in *Griggs v. Duke Power Co.* (1971); however, there have been a number of subsequent court cases challenging the extent to which disparate impact is cognizable under ECOA. These issues are outside the scope of this mini review.

6 See 12 CFR § 1,002.9. The term “adverse action” is defined generally to include a “refusal to grant credit in substantially the amount or on substantially the terms requested” by an applicant or a termination or other unfavorable change in terms on an existing account. 12 CFR § 1,002.2 (c). Similar requirements for notices exist in the E. U., although the requirement appears to be less stringent in some instances. For example, FCRA adverse action notices apply to new and existing credit lines, while the E. U. Consumer Credit Directive applies only to newly extended credit. Moreover, commentators continue to debate the nuances of whether and how the GDPR provides consumers a right to explanation for decisions made by ML models.

7 See 12 CFR § 1,002.9(b) and the Official Commentary thereto included in Supplement I of Regulation B.

8 In the text of this mini review, the authors use early definitions of interpretability and explainability that have become accepted trade jargon. However, the authors also note important work initiated by the United States National Institute of Standards and Technology (NIST) that will likely shape these definitions in future years (Phillips et al., 2020; Broniatowski 2021). In particular, Broniatowski’s work based in Fuzzy-Trace Theory gives perhaps the most authoritative treatment to date of the fundamental differences between interpretability, or high-level contextualization based on purpose, values, and preferences, versus low-level technical explanations.

9 See Interagency Guidance on Model Risk Management (Federal Reserve Board, SR Letter 11–7).

10 See also Twenty-one Fairness Definitions and Their Politics.

11 See Consumer Financial Protection Bureau, *Supervisory Highlights*, Issue 9, Fall 2015, p. 29.

12 See Part 1,607—Uniform Guidelines on Employee Selection Procedures (1978) § 1,607.4.

13 We cite these specific references because they have influenced academic and popular debates about algorithmic discrimination and are relevant for predictive modeling practitioners in general. However, these techniques sometimes fail to meet the requirements set forth by applicable regulations in fair lending as addressed in the following section.

14 E.g., aequitas and H2O Driverless AI.

15 E.g., see, New Patent-Pending Technology from Equifax Enables Configurable AI Models; see also, Deep Insights into Explainability and Interpretability of Machine Learning Algorithms and Applications to Risk Management.

16 See comparisons of EBMs with other interpretable and black-box models by Microsoft Research: https://github.com/interpretml/interpret.

17 Monotonic GBMs, as implemented in XGBoost or H2O.

18 See Explaining Measures of Fairness with SHAP, created by Scott Lundberg, the author of several authoritative works on Shapley values.

19 United States provisional patent 63/153,692 is associated with technologies implemented to derive Figure 1.

20 See The Official Commentary of 12 CFR § 1,002.9 (b) (2) for Regulation B.
